# Adapting AlphaLISA high throughput screen to discover a novel small-molecule inhibitor targeting protein arginine methyltransferase 5 in pancreatic and colorectal cancers

**DOI:** 10.18632/oncotarget.18102

**Published:** 2017-05-23

**Authors:** Lakshmi Prabhu, Han Wei, Lan Chen, Özlem Demir, George Sandusky, Emily Sun, John Wang, Jessica Mo, Lifan Zeng, Melissa Fishel, Ahmad Safa, Rommie Amaro, Murray Korc, Zhong-Yin Zhang, Tao Lu

**Affiliations:** ^1^ Department of Pharmacology and Toxicology, Indiana University School of Medicine, Indianapolis, IN, USA; ^2^ Chemical Genomics Core Facility, Indiana University School of Medicine, Indianapolis, IN, USA; ^3^ Department of Biochemistry and Molecular Biology, Indiana University School of Medicine, Indianapolis, IN, USA; ^4^ Department of Medicinal Chemistry and Molecular Pharmacology, Purdue University, West Lafayette, IN, USA; ^5^ Department of Chemistry and Biochemistry, University of California, San Diego, La Jolla, CA, USA; ^6^ Department of Pathology and Laboratory Medicine, Indiana University School of Medicine, Indianapolis, IN, USA; ^7^ Department of Medicine, Indiana University School of Medicine, Indianapolis, IN, USA; ^8^ Department of Medical and Molecular Genetics, Indiana University School of Medicine, Indianapolis, IN, USA; ^9^ Department of Pediatrics, Wells Center for Pediatric Research, Indiana University School of Medicine, Indianapolis, IN, USA

**Keywords:** AlphaLISA, colorectal cancer, pancreatic ductal adenocarcinoma, PRMT5, small-molecule inhibitor

## Abstract

Pancreatic ductal adenocarcinoma (PDAC) and colorectal cancer (CRC) are notoriously challenging for treatment. Hyperactive nuclear factor κB (NF-κB) is a common culprit in both cancers. Previously, we discovered that protein arginine methyltransferase 5 (PRMT5) methylated and activated NF-κB. Here, we show that PRMT5 is highly expressed in PDAC and CRC. Overexpression of PRMT5 promoted cancer progression, while shRNA knockdown showed an opposite effect. Using an innovative AlphaLISA high throughput screen, we discovered a lead compound, PR5-LL-CM01, which exhibited robust tumor inhibition effects in both cancers. An *in silico* structure prediction suggested that PR5-LL-CM01 inhibits PRMT5 by binding with its active pocket. Importantly, PR5-LL-CM01 showed higher anti-tumor efficacy than the commercial PRMT5 inhibitor, EPZ015666, in both PDAC and CRC. This study clearly highlights the significant potential of PRMT5 as a therapeutic target in PDAC and CRC, and establishes PR5-LL-CM01 as a promising basis for new drug development in the future.

## INTRODUCTION

Gastrointestinal cancer refers to malignant conditions of the gastrointestinal tract (GI tract) and accessory organs of digestion. Among them, pancreatic ductal adenocarcinoma (PDAC) and colorectal cancer (CRC) are two of the most challenging cancer types. For instance, PDAC has a very poor prognosis with a median survival between 6-8 months [1, 2] and ~8% of 5-year survival rate. According to the American Cancer Society, PDACs represent the majority of the pancreatic cancer cases. The available options for treatment are hardly adequate in improving the quality of life of PDAC patients. Current therapy mainly consists of surgery, if diagnosed in the early stages. In more advanced stages, combined local treatment of radiation therapy and drugs such as gemcitabine plus nab-paclitaxel or FOLFIRINOX (combination of 5-flurouracil, leucovorin, irinotecan and oxaliplatin) are used [3]. However, in addition to having a number of side effects, these therapeutic strategies barely increase the life span of the patient and provide little chance for a cure.

Equally devastating, CRC is the second leading cause of death in men and women combined in the United States [4]. It results from a series of genetic and epigenetic changes in colon epithelial cells, with successive mutations that accumulate over time [5]. Despite important advances in recent years, more than 40% of CRC patients will experience disease recurrence following primary therapy. The fact that there is currently “no cure” for a significant portion of patients with metastasis at the time of diagnosis creates a large medical need and an urgent demand for the discovery of effective therapeutic targets and new medicine for CRC treatment.

An important common feature of both PDAC [6–8] and CRC [9–11] is the hyperactive nuclear factor κB (NF-κB) activity [12]. NF-κB is a critical eukaryotic transcription factor whose family consists of five members: RelA (p65), RelB, cRel, NF-κB1 (p50 and precursor p105), and NF-κB2 (p52 and precursor p100) [13]. Among these, the p65:p50 heterodimer is a prototype. NF-κB signaling can be classified into canonical and non-canonical pathways. The canonical pathway has been well established as a key contributor to development of both PDAC [14, 15] and CRC [16, 17]. In this pathway, the inhibitor of κB (IκBα) sequesters the p65:p50 heterodimer in an inactive state in the cytoplasm. Upon receiving extracellular signals such as pro-inflammatory cytokines, *etc*., IκB kinase phosphorylates IκBα, leading to its degradation, the release of the p65:p50 complex, and the activation of NF-κB target genes [18]. A number of these downstream NF-κB target genes have been implicated in cancer. Increased NF-κB activation is not only associated with poor disease prognosis, but also with developing resistance against chemotherapy in PDAC and CRC [19, 20]. Thus, controlling NF-κB activity is critical to the treatment of PDAC and CRC.

Recently, we became the first to discover that the protein arginine methyltransferase 5 (PRMT5) serves as a potent activator of NF-κB *via* dimethylating arginine 30 of its p65 subunit [21]. The nature of PRMT5 as a histone modifying enzyme makes it an important therapeutic target to pursue. As a member of the family of PRMTs, PRMT5 methylates arginine residues by catalyzing the transfer of methyl groups from a ubiquitous co-factor—S-adenosyl-l-methionine (SAM) to specific arginine residues on their target proteins, leading to the symmetric dimethylation of its substrates [22–24].

In these studies we provide the first evidence that PRMT5 is highly overexpressed in PDAC and CRC. Overexpression of PRMT5 promoted cancer progression through increased activation of NF-κB, cell growth, anchorage-independent growth and cell migration in both PDAC and CRC. Importantly, we developed a PRMT5-specific AlphaLISA HTS technique (Amplified luminescent proximity homogeneous assay-linked immunosorbent assay high throughput screen) by which we identified a novel small-molecule inhibitor of PRMT5, PR5-LL-CM01. We showed that PR5-LL-CM01 is much more potent than the only commercial PRMT5 inhibitor, EPZ015666 [25]. Treatment with PR5-LL-CM01 led to a decrease in the activation of NF-κB and tumor progression in both PDAC and CRC. Using *in silico* modeling, we proposed that PR5-LL-CM01 inhibits the activity of PRMT5 by direct binding to the glutamate 444 (Glu444) residue in the active pocket of PRMT5 [26].

Here, we define for the first time a significant role of PRMT5 as a tumor promoter in PDAC and CRC. By discovering our leading compound PR5-LL-CM01 with the AlphaLISA HTS technique, we proved that PRMT5 is a promising therapeutic target in PDAC and CRC. Of extreme importance and significance, PR5-LL-CM01 could serve as an important basis for new drug development in PDAC and CRC.

## RESULTS

### PRMT5 is overexpressed in PDAC and CRC

**Figure 1:**
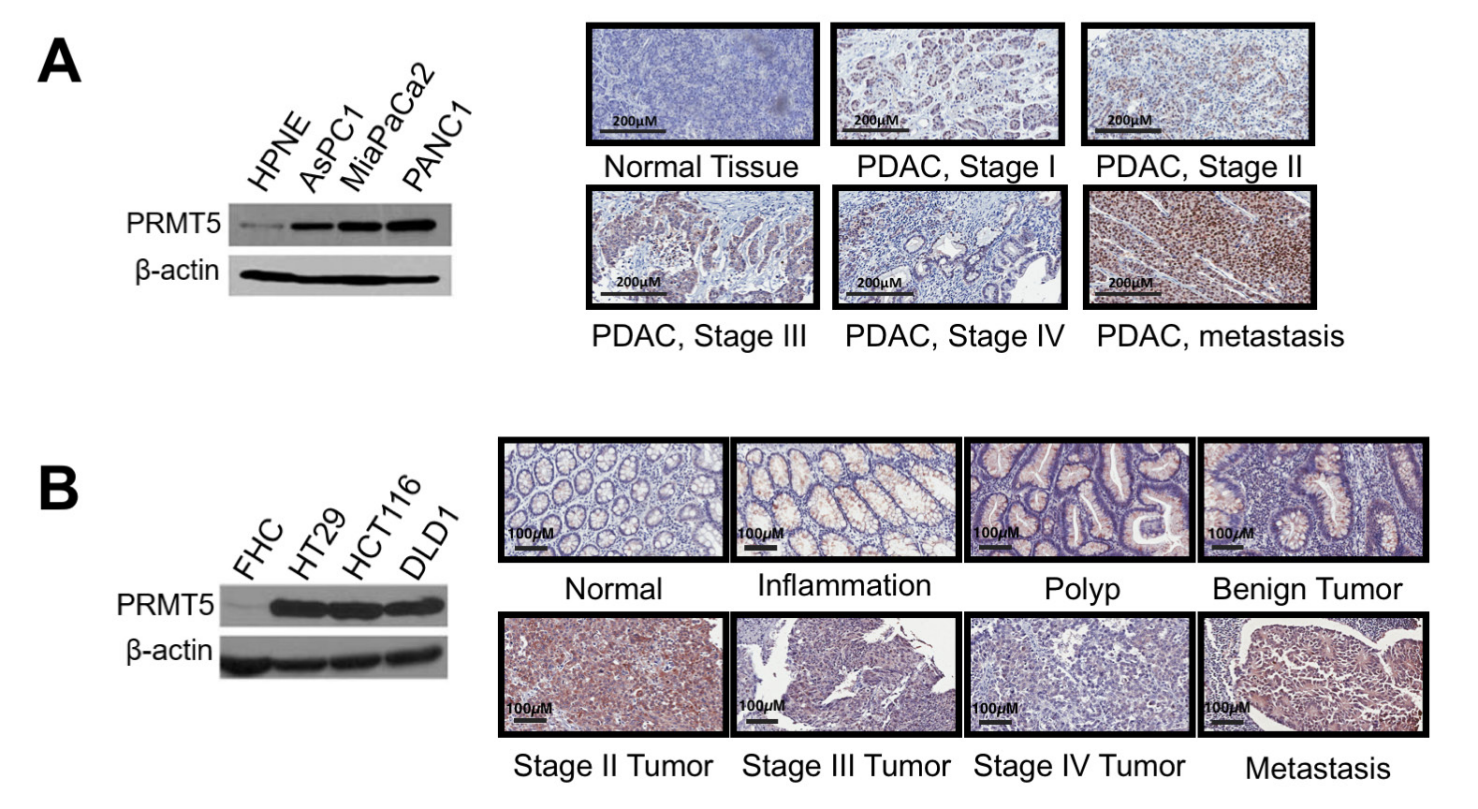
PRMT5 is overexpressed in pancreatic and colorectal cancer **A**. and **B**. (Left panels), Western blots, showing that **A**. PRMT5 expression is higher in PDAC cells (AsPC1, MiaPaCa2 and PANC1) as compared to control pancreatic cells HPNE. **B**. PRMT5 expression is higher in CRC cells (HT29, HCT116 and DLD1) as compared to control colon cells FHC. β-actin was used as a loading control. **A**. and **B**. (Right panels), IHC staining, showing that **A**. PRMT5 expression was higher in PDAC tumor tissues as compared to the normal tissue. **B**. PRMT5 protein expression was higher in the inflammation, polyp, advanced stages of CRC and the metastatic stage as compared to the normal colon tissue.

In order to determine whether PRMT5 is a tumor promoter in PDAC and CRC, we first examined PRMT5 expression in both cancers by Western blotting and immunohistochemistry (IHC) assay. As shown in Figure 1A (left panel), PRMT5 was highly expressed in PDAC cells (AsPC1, MiaPaCa2 and PANC1) as compared to human normal pancreas HPNE cells. Tissue microarray (TMA) was further detected for PRMT5 expression using IHC assay. We showed that PRMT5 expression was significantly higher in various stages of PDAC, and particularly in the metastatic stage, as compared to the normal PDAC adjacent tissue (Figure 1A, right panel, and Supplementary Figure S1A). Similar experiments were carried out in a series of CRC cells (HT29, HCT116 and DLD1), with normal colon FHC cells as the control. Again, we observed that PRMT5 is highly expressed in CRC cells (Figure 1B, left panel). Moreover, IHC assay of CRC TMA also indicated that PRMT5 had much higher expression in samples ranging from inflammation, polyp, to the metastatic stage of CRC as compared to adjacent normal tissue (Figure 1B, right panel, and Supplementary Figure S1B). These data clearly demonstrated that PRMT5 is substantially overexpressed in both PDAC and CRC.

### PRMT5 promotes cell proliferation, anchorage-independent growth and cell migration in PDAC and CRC cells

**Figure 2:**
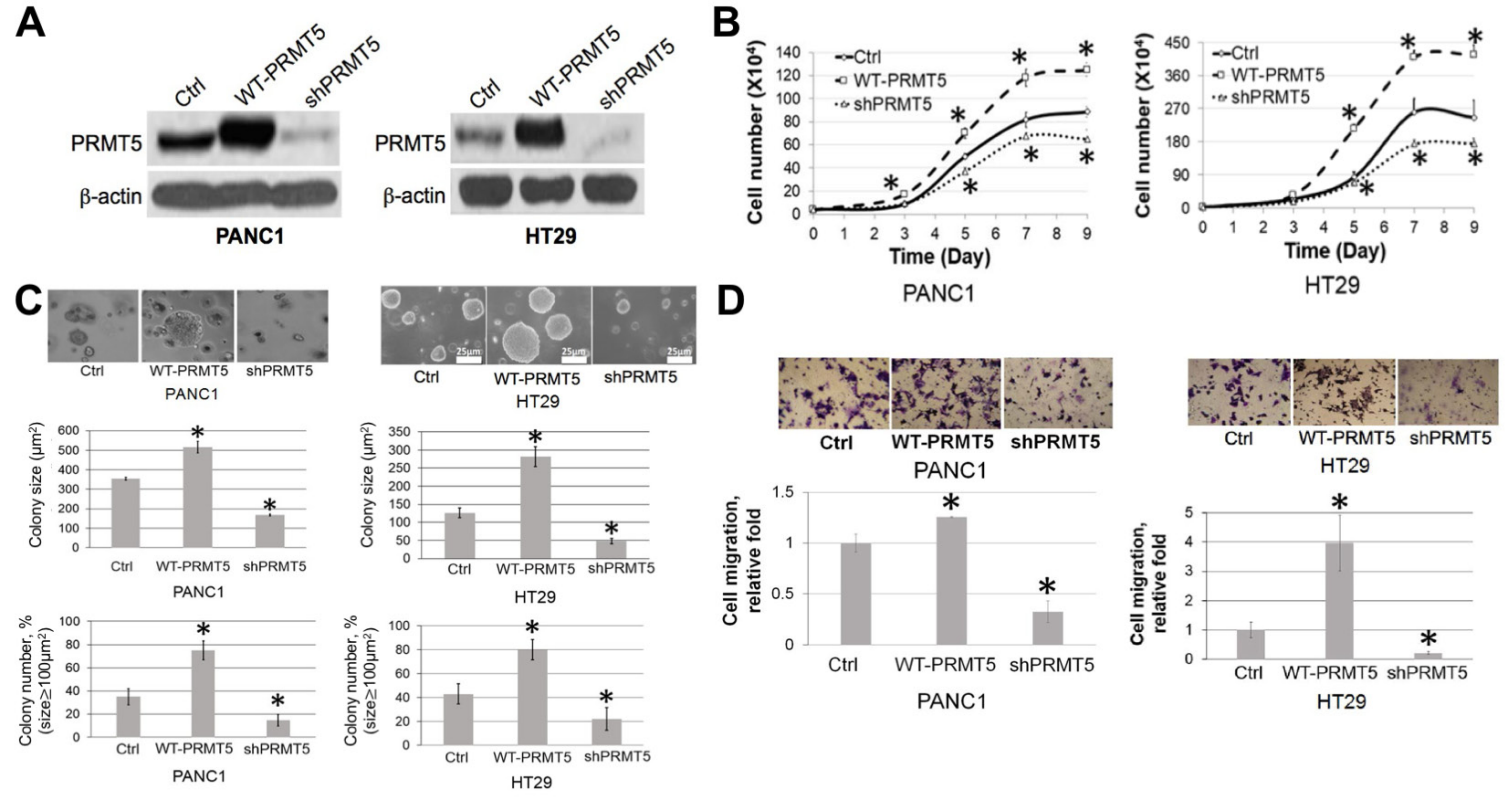
PRMT5 is important for cell proliferation, anchorage-independent growth and migration of PDAC and CRC **A**. Western blot, confirming stable PRMT5 overexpression and shPRMT5 knockdown in PANC1 and HT29 cells. **B**. Cell proliferation assay, showing that cell proliferation was significantly higher in the WT-PRMT5 cell lines, while shPRMT5 cells exhibited quite opposite effect in both PANC1 and HT29 cell lines. **C**. Anchorage-independent growth assay, showing that anchorage-independent growth was significantly higher in the WT-PRMT5 cell lines, while dramatically reduced in the shPRMT5 cells. Both colony size and number are quantified at the bottom of the corresponding panels. The data represent the means ± standard deviation (S.D.) for three independent experiments. *P < 0.05 *vs*. control (Ctrl) group. **D**. Cell migration assay, showing that cell migration was significantly higher in the WT-PRMT5 overexpression cells, while significantly reduced in the shPRMT5 cells. Upper panels, representative pictures in 20X magnification. Lower panel, quantification for the change in migration. The data represent the means ± S.D. for three independent experiments. *P < 0.05 *vs*. Ctrl.

Since PRMT5 is highly expressed in PDAC and CRC, we wondered whether it serves as a tumor promoter in these cancers. In order to test this possibility, we examined the effect of PRMT5 on various characteristics of cancer cells including cell proliferation, anchorage-independent growth, and cell migration. Stable cells with either PRMT5 overexpression or shRNA knockdown were established in PANC1 (PDAC) and HT29 (CRC) cells. Western blotting was conducted to confirm the expected PRMT5 expression in these cells (Figure 2A). Using these cells, we further showed that overexpression of PRMT5 promoted cell growth, while shRNA knockdown reduced this effect in both PDAC and CRC cells (Figure 2B), strongly suggesting that PRMT5 is the promoter for cell proliferation in these cells.

Moreover, we conducted an anchorage-independent growth assay in soft agar. Data suggested that PRMT5 overexpression led to a dramatic increase in both the colony size and number in PANC1 and HT29 cells (Figure 2C), while shPRMT5 knockdown significantly reduced this ability, confirming the critical role that PRMT5 plays in promoting anchorage-independent cell growth in PDAC and CRC.

Another well-known property of cancer cells is the strong migration ability, which is critical for tumor invasion and metastases [31]. As observed in Figure 2D, Boyden chamber assays were conducted, demonstrating that overexpression of PRMT5 substantially increased the number of migrated cells, whereas shPRMT5 knockdown showed quite opposite effect, pointing out the pivotal role that PRMT5 plays in the migratory ability of both PDAC and CRC.

Collectively, the above data provided strong evidence regarding the overall tumor promoting ability of PRMT5 in both PDAC and CRC.

### Using the innovative AlphaLISA HTS screen to discover a novel PRMT5 inhibitor PR5-LL-CM01

**Figure 3:**
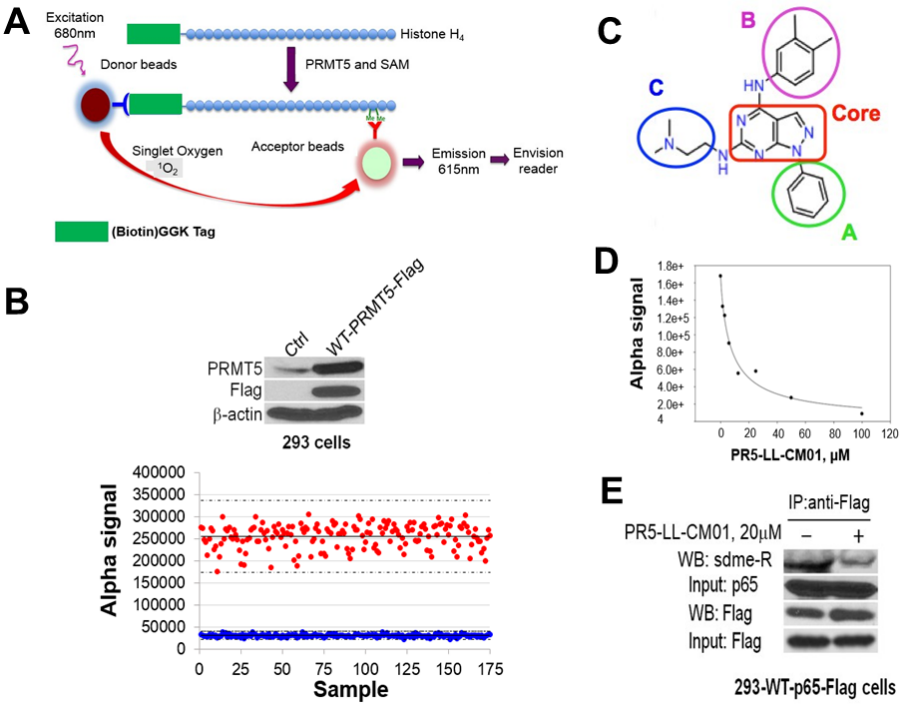
Discovery of PR5-LL-CM01 as a novel PRMT5 inhibitor through PRMT5-specific AlphaLISA HTS technique **A**. Schematic illustration of the AlphaLISA technique. Biotinylated histone H4 is incubated with PRMT5 and methyl donor SAM. PRMT5 symmetrically dimethylates H4 on its arginine 3, a product that is recognized by Acceptor beads. Following the binding of Donor beads, the chemiluminescent signal is emitted and detected by the EnVision^®^ Reader. The intensity of the Alpha signal is proportional to the PRMT5 activity. **B**. *(Upper panel*) Western blot, showing Flag-tagged WT-PRMT5 was overexpressed in the 293-WT-PRMT5-Flag cells as compared to the control 293 cells. 293-WT-PRMT5-Flag cells were used to purify PRMT5 for AlphaLISA assay. (*Lower panel)* Z’-plot of AlphaLISA assay, showing that Alpha signal with PRMT5 (red dots) is significantly higher than those without PRMT5 (blue dots). The mean values are represented with solid horizontal lines in the center of each sample group. Z’ value for our experiment was 0.6. **C**. Structure of PR5-LL-CM01, highlighting the core structure of a pyrozolo-pyrimidine group (red box), and three surrounding groups A, B and C, respectively. **D**. Calculation of IC_50_ of PR5-LL-CM01 using AlphaLISA, showing PR5-LL-CM01 concentration-dependent decrease of Alpha signal. The IC_50_ was ~7.5 μM. **E**. Co-IP-Western blot, showing that treatment with 20μM PR5-LL-CM01 inhibited p65 methylation, a PRMT5 substrate, in 293-WT-p65-Flag cells. Flag beads were used to pull down WT-p65-Flag and samples were probed with anti-symmetric dimethyl arginine motif Ab (sdme-RG Ab).

Based on the above observations, we hypothesize that inhibition of PRMT5 with small-molecule inhibitors has potential therapeutic implications in PDAC and CRC. In order to successfully perform the HTS screen, we adapted the AlphaLISA technique (PerkinElmer) into an assay that could precisely quantify PRMT5 methylation of its substrate in a 384-well HTS platform. Compared to the conventional methods such as time resolved fluorescence energy transfer (TR-FRET) assay, the AlphaLISA technology is proved to be more sensitive, reliable, and adaptable for performing large-scale reactions. Thus it proves to be a more robust assay to be used for screening as compared to other methods used in the past [32, 33]. To date, AlphaLISA has never been used for screening of PRMT5 inhibitors in the HTS format. We have overcome obstacles to successfully modify this technique from bench scale to a condition that can provide robust results in a robotic system. As shown in Figure 3A, PRMT5 transferred the methyl groups from its methyl donor SAM to biotin-histone H4 peptide, leading to its symmetric dimethylation, which was recognized by the Acceptor beads conjugated with the anti-H4R3me2 antibody and the streptavidin tagged Donor beads. Upon excitation with light, emitted signal was detected by an EnVision^®^ Reader, which was proportional to the amount of dimethylated H4R3. Therefore, for any small-molecule inhibitor used to inhibit the activity of PRMT5, a reduced signal emission would be detected.

The PRMT5 enzyme used in our screen was purified by conducting co-immunoprecipitation with anti-Flag beads in a 293-PRMT5-Flag cell line established by us (Figure 3B, top panel). This enzyme prep is highly active, with a near 7-fold increase of Alpha signal as compared to that of negative control (Figure 3B, bottom panel). Z’ factor, a statistical parameter for the robustness of the AlphaLISA assay, was calculated to be 0.6 (Figure 3B, bottom panel). An assay with a Z’ value > 0.5 is widely considered to be sensitive enough for a successful HTS [34]. Therefore, this test confirmed that our adapted PRMT5-specific AlphaLISA HTS technique was sensitive, robust, and successful.

We screened a library with 10,000 small molecules. A representative 384-well plate in the HTS (Supplementary Figure S2) shows a potential hit highlighted in red, with a significant decrease observed in the AlphaLISA signal, as compared to that with no inhibitor. Several top hits were identified and confirmed using both AlphaLISA and MTT assay in PDAC and CRC cells. Among these, the leading compound was PR5-LL-CM01. PR5-LL-CM01 consists of a pyrozolo-pyrimidine core and three peripheral A, B and C groups (Figure 3C). We confirmed that the IC_50_ of PR5-LL-CM01 was 7.5 μM by the AlphaLISA approach (Figure 3D). Furthermore, in order to check the specificity of PR5-LL-CM01 to PRMT5, we generated PR5-LL-IEC01, a structural analog of PR5-LL-CM01 (Supplementary Figure S3A). The IC_50_ of PR5-LL-IEC01 was calculated to be 118μM by AlphaLISA (Supplementary Figure S3B), about 16-fold higher than that of PR5-LL-CM01 (Figure 3D), affirming that the inhibition of PRMT5 methyltransferase activity observed by AlphaLISA assay was specific to small-molecule inhibitor PR5-LL-CM01.

Moreover, we examined the inhibition by PR5-LL-CM01 against other PRMT family members using the HotSpot radioisotope-based platform (Reaction Biology Corp) [35]. As shown in Supplementary Figure S4, these PRMTs showed zero effect, or at least a 10-fold higher IC_50_ than that of PRMT5, indicating the high specificity of PR5-LL-CM01 for PRMT5.

We previously found that PRMT5 activated NF-κB through methylation of its p65 subunit [21], therefore, we sought to determine whether treatment with PR5-LL-CM01 would reduce p65 methylation. Accordingly, 293 cells engineered to overexpress Flag-p65 were treated with 20μM PR5-LL-CM01 for 24hrs. Flag-p65 was pulled down with anti-Flag-M2 beads and analyzed with Western blot by probing with anti-dimethylated arginine antibody (Figure 3E). Compared to the untreated control cells, treatment with PR5-LL-CM01 significantly inhibited PRMT5-mediated p65 methylation. Collectively, the above experiments demonstrated that we have successfully developed a PRMT5-specific AlphaLISA HTS technique by which we discovered a novel PRMT5 inhibitor, PR5-LL-CM01.

### PR5-LL-CM01 is a potent inhibitor of PRMT5 *in vitro*

**Figure 4:**
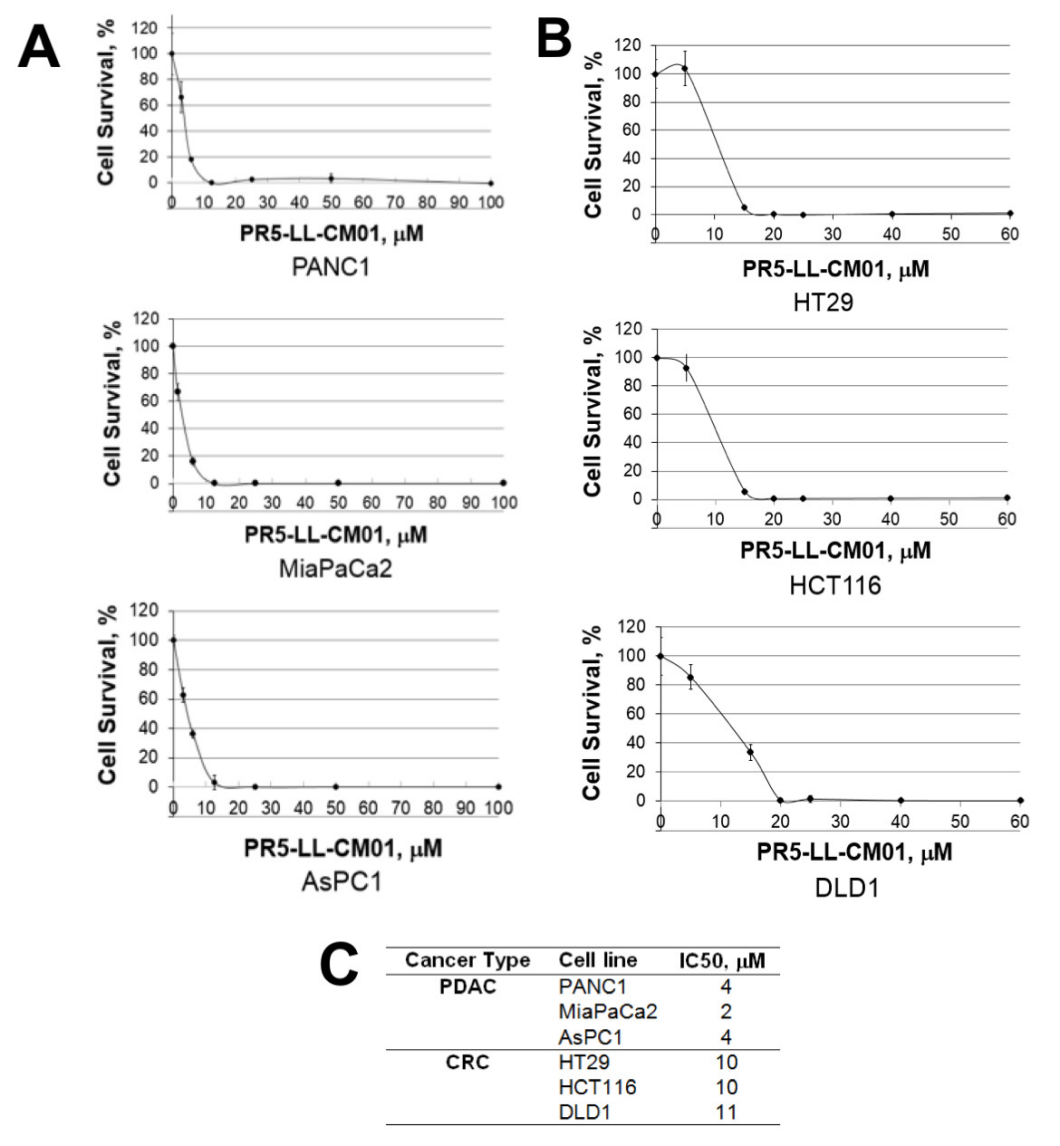
PR5-LL-CM01 is a potent inhibitor of PRMT5 in PDAC and CRC cells **A**. MTT assay in PDAC cells (PANC1, MiaPaCa2 and AsPC1), showing that cell viability decreased significantly in presence of increasing concentrations of PR5-LL-CM01. **B**. MTT assay in CRC cells (HT29, HCT116, and DLD1), showing dramatic decrease of cell viability in presence of increased concentrations of PR5-LL-CM01. **C**. Table, summarizing the IC_50_ values for PR5-LL-CM01 in PDAC and CRC cells, respectively.

In order to determine the efficacy of PR5-LL-CM01, we used both PDAC and CRC cells as the *in vitro* models. Recently, Chan-Penebre *et al.* identified a PRMT5 inhibitor, EPZ015666 and showed its high efficacy in inhibiting PRMT5 in mantle cell lymphoma disease models [25]. We wondered whether EPZ015666 would be effective in PDAC and CRC. Both PDAC and CRC cells were treated with increasing concentrations of PR5-LL-CM01 or EPZ015666, and quantified for cell viability with the MTT assay. We showed that PR5-LL-CM01 had a range of IC_50_ at 2-4 μM in PDAC cells (PANC1, MiaPaCa2 and AsPC1) (Figure 4A, 4C), and a range of IC_50_ at 10-11 μM in CRC cells (HT29, HCT116 and DLD1) (Figure 4B, 4C). However, EPZ015666 was much less effective as compared to PR5-LL-CM01, with a range of IC_50_ at 50-95 μM for PDAC cells, and a range of IC_50_ at 180-195 μM for CRC cells (Supplementary Figure S5). Therefore, our data suggested that PR5-LL-CM01 is a more potent PRMT5 inhibitor than EPZ015666 in PDAC and CRC cells.

Additionally, as compared to cancer cell lines, the respective normal control cells had a much higher survival at the IC_50_ observed in their cancer cell line counterparts (Supplementary Table 1). HPNE and FHC are the normal pancreatic and colon control cell lines respectively, that were used in the present study. This suggested that PR5-LL-CM01 had higher efficacy to specifically inhibit cancer cells and demonstrated low toxicity in normal cells.

In addition, we determined the IC_50_ of the inactive structural analog PR5-LL-IEC01 in PANC1 and HT29 cells using the MTT assay. Not surprisingly, we observed remarkably less potency of PR5-LL-IEC01 to decrease the cell viability in these lines, with an IC_50_ of over 14-40-fold higher (Supplementary Figure S6) than that of PR5-LL-CM01 (Figures 4A and 4B) in the same cell lines.

Next, we used the anchorage-independent assay described previously to check if treatment with PR5-LL-CM01 affected colony formation of either PDAC or CRC cells. We observed that treatment with PR5-LL-CM01 strongly inhibited colony forming ability in both PANC1 and HT29 cells (Supplementary Figure S7).

In light of recent work suggesting 3D spheroidal cell culture may closely mimic the *in vivo* tumor microenvironment such as having a hypoxic core, decreased cell-cell contact and greater survival [36, 37], we determined the efficacy of PR5-LL-CM01 against 3D spheroids of PDAC and CRC cells (Supplementary Figure S8). With increasing concentrations of PR5-LL-CM01, we observed a dosage-dependent decrease in the ability of both PANC1 and HT29 cells to form 3D spheroids in culture, highlighting the tumor-inhibiting potential of PR5-LL-CM01 *in vitro*.

### PR5-LL-CM01 inhibited NF-κB activation and its target gene expression in PDAC and CRC cells

**Figure 5:**
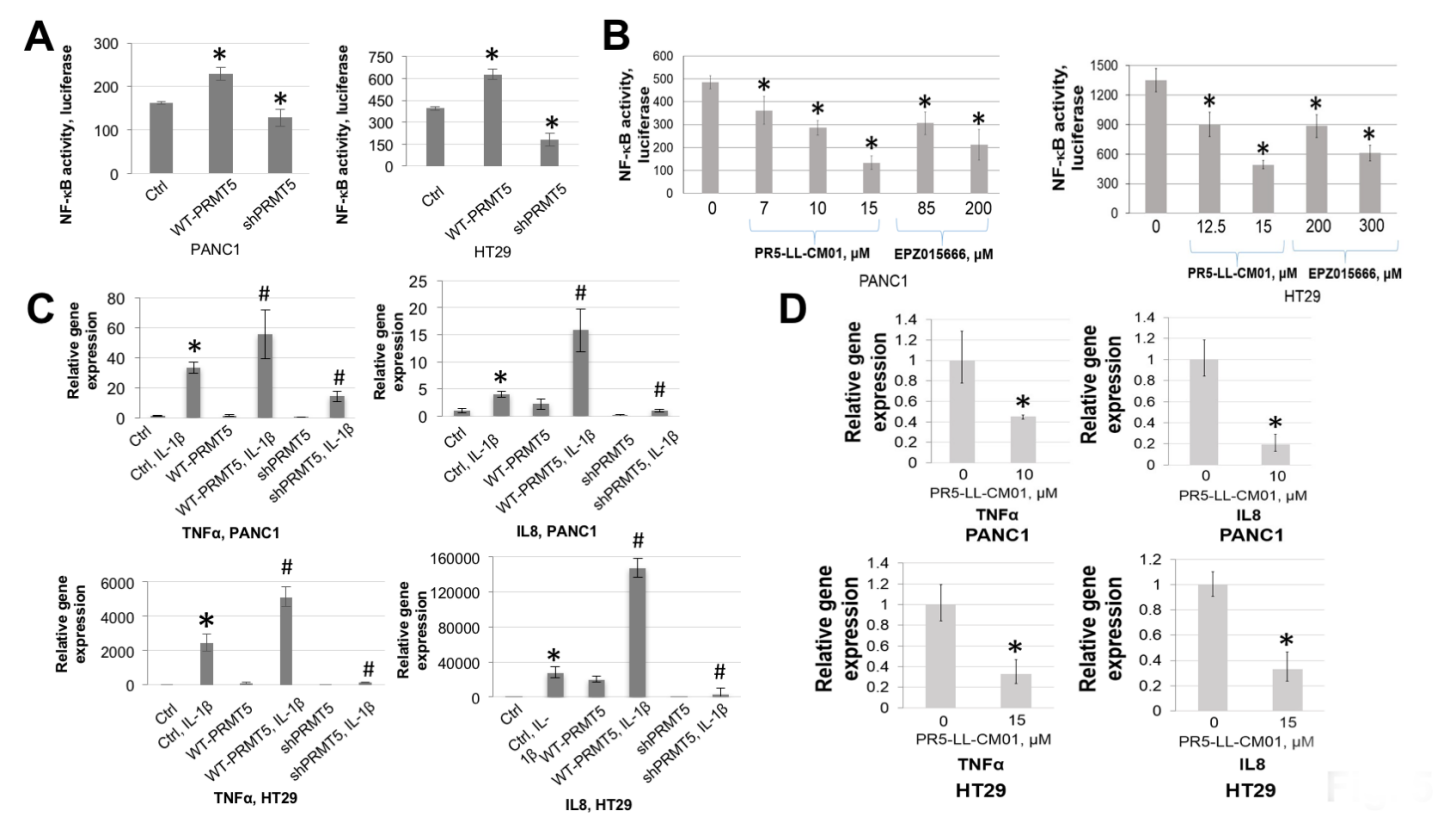
Treatment with PR5-LL-CM01 significantly inhibited NF-κB activation in PDAC and CRC cells **A**. NF-κB luciferase assay, showing that overexpression of WT-PRMT5 led to NF-κB activation, while shPRMT5 resulted in quite opposite effect in both PANC1 and HT29 cells. *P < 0.05 *vs.* Ctrl. **B**. Luciferase assay, showing a decrease in NF-κB activation with increasing concentrations of PR5-LL-CM01 in PANC1 (left panel) and HT29 cells (right panel). A much higher concentration of EPZ015666 is needed in order to reach similar level of NF-κB inhibition as that of PR5-LL-CM01. The data represent the means ± S.D. for three independent experiments. *P < 0.05 *vs*. Untreated group. **C**. qPCR analysis, showing that overexpression of PRMT5 significantly enhanced IL-1β-triggered NF-κB target genes (TNFα and IL8) expression, while shPRMT5 exhibited quite opposite effect, in both PANC1 and HT29 cells. *P < 0.05 *vs*. Ctrl; ^#^*P* < 0.05 *vs*. Ctrl+IL-1β-treated group. **D**. qPCR analysis, showing that treatment with PR5-LL-CM01 dramatically decreased TNFα and IL8 expression, in both PANC1 and HT29 cells. The data represent the means ± S.D. for three independent experiments. *P < 0.05 *vs*. Untreated group.

Previously, we discovered that PRMT5 activates NF-κB through methylation of its p65 subunit in HEK293 cells [21]. In Figure 5A, by using a κB luciferase assay, we confirmed that overexpression of PRMT5 significantly enhanced NF-κB activity, while shPRMT5 knockdown exhibited a quite opposite effect. Next, we determined the effect PR5-LL-CM01 on NF-κB activation. As shown in Figure 5B, treatment with increasing concentrations of PR5-LL-CM01 resulted in a corresponding decrease in NF-κB activation in PANC1 and HT29 cells. In great contrast, a much higher concentration of EPZ015666 was required to observe a similar effect (Figure 5B), demonstrating the much higher efficacy of PR5-LL-CM01 to decrease the NF-κB activation in PDAC and CRC cells*.* Moreover, treatment with inactive analog PR5-LL-IEC01 had no significant effect on the NF-κB activation in both PDAC and CRC cells, indicating the specificity of PR5-LL-CM01 to decrease NF-κB activation in these cell lines (Supplementary Figure S9).

We previously showed that PRMT5-mediated NF-κB activation led to the induction of typical NF-κB target genes, such as tumor necrosis α (TNFα) and interleukin 8 (IL8) in 293 cells. We therefore wondered whether an upregulation of these genes is observed in PDAC and CRC cells. Quantitative PCR showed that upon stimulation with IL-1β, there was a substantial increase in the expression of TNFα and IL8 in PRMT5 overexpressing cells, while a dramatic reduction was observed upon shPRMT5 knockdown, in both PANC1 and CRC cells (Figure 5C). Treatment with PR5-LL-CM01 also led to a significant decrease in both genes (Figure 5D), thus indicating that PR5-LL-CM01 decreased the PRMT5-mediated NF-κB-dependent gene activation. Overall, we demonstrated that PR5-LL-CM01 had significant efficacy in inhibiting NF-κB activation and its downstream gene expression in PDAC and CRC cells.

### PR5-LL-CM01 has dramatic anti-tumor efficacy *in vivo*

**Figure 6:**
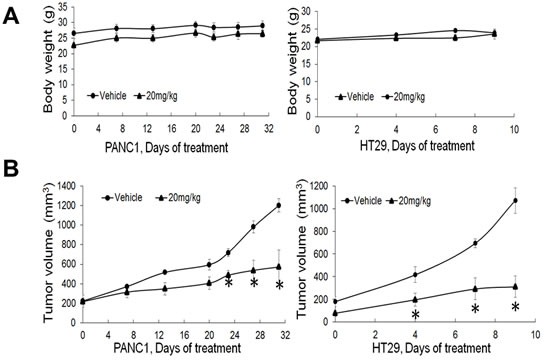
PR5-LL-CM01 displayed significant anti-tumor effect *in vivo* **A**. No significant changes in body weight were observed over the course of treatment in either PANC1 or HT29 model after treatment with 20mg/kg of PR5-LL-CM01. (*P < 0.05, *n* = 4). **B**. Tumor efficacy study for PANC1 or HT29 cells which were subcutaneously implanted in NSG mice. Inhibition of tumor growth was observed upon treatment with 20mg/kg of PR5-LL-CM01 intraperitoneally for 3X/week, as compared to the vehicle control. (*P < 0.05, *n* = 4).

Since we have shown that PR5-LL-CM01 had great anti-cancer efficacy in PDAC and CRC cells *in vitro*, we wonder whether PR5-LL-CM01 exhibits tumor inhibition effect *in vivo*. Either PANC1 or HT29 cells were subcutaneously xenografted into NOD scid gamma (NSG) mice and then treated with PR5-LL-CM01 three times per week at 20mg/kg till the day the mice were sacrificed. Both body weight and tumor size were monitored during this process. As shown in Figure 6A, injection of PR5-LL-CM01 did not visibly affect the mice body weight. However, it led to significant tumor inhibition in both PANC1 and HT29 (Figure 6B) xenografted mice, demonstrating the strong anti-tumor efficacy of PR5-LL-CM01 in both PDAC and CRC.

### Structural modeling of binding interactions between PR5-LL-CM01 and PRMT5

**Figure 7:**
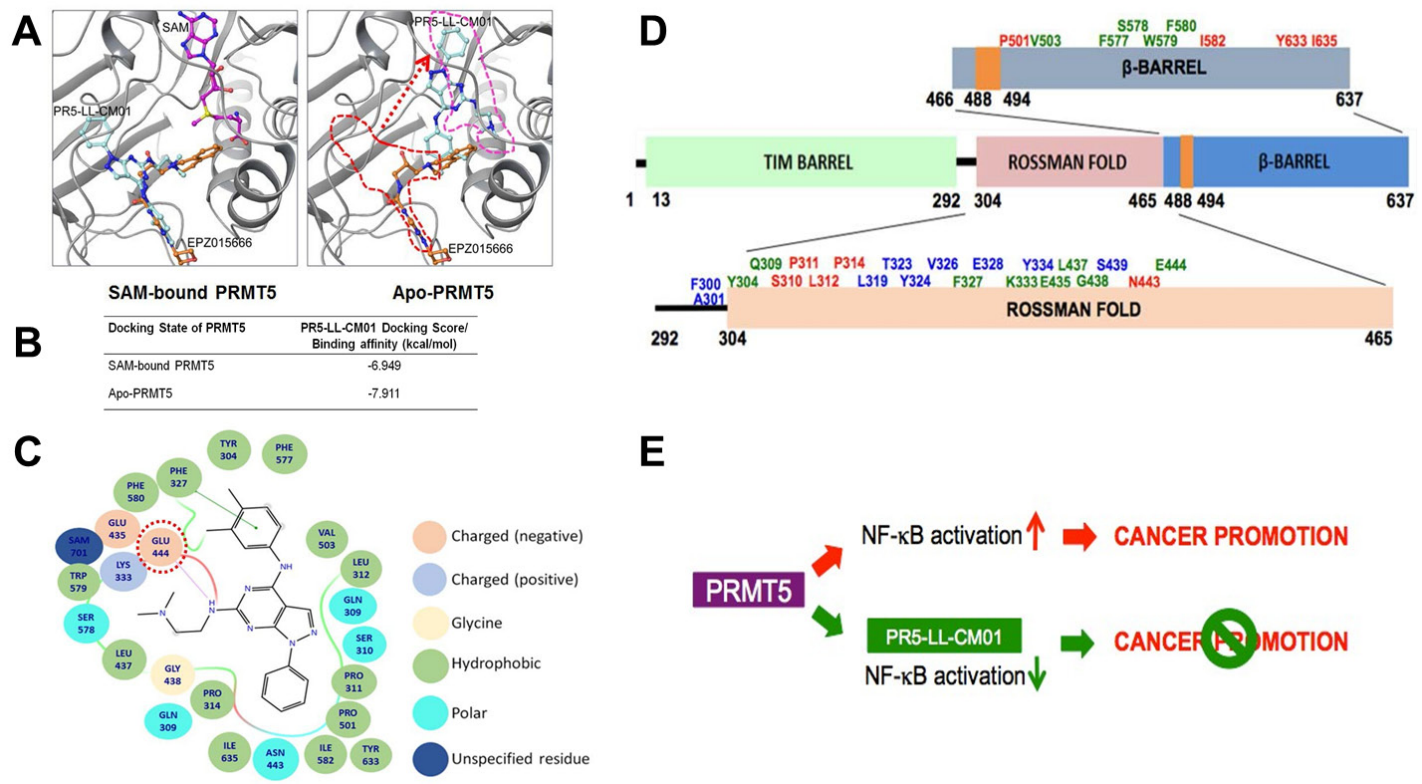
*In silico* prediction of the binding of PR5-LL-CM01 and EPZ015666 to PRMT5 **A**. *left panel*, In SAM (purple color)-bound condition, the binding sites for PR5-LL-CM01 (turquoise color) and EPZ015666 (orange color) on PRMT5 overlap to a great extent, suggesting that these two compounds inhibit PRMT5 through largely similar group of binding sites. *Right panel*, in Apo-PRMT5 condition, the binding of EPZ015666 remains the same as that in the *left panel*, however, PR5-LL-CM01 has shifted dramatically from its *left panel’s* position (red dotted line) to the new position (pink dotted line), which is same as SAM's position in the *left panel*. **B**. Table, listing the binding affinities of PR5-LL-CM01-PRMT5 interactions in SAM bound and Apo-PRMT5 conditions. PR5-LL-CM01 showed favorable binding energies under both conditions, with Apo-PRMT5 condition having a slightly stronger binding affinity (-7.911 kcal/mol). **C**. Ligand affinity map in the SAM-bound condition, depicting the PRMT5 residues interacted with PR5-LL-CM01. Different bonds or charges are symbolized on the right side of the figure. Key site, Glu444, is circled in red dotted line. **D**. Binding residue distribution diagram, illustrating the PRMT5 residues that potentially bind to PR5-LL-CM01 alone (red font), EPZ015666 alone (blue font), or both (green font). **E**. Hypothetical model, proposing that PRMT5 overexpression promotes cancer phenotype, whereas inhibition using PR5-LL-CM01 impedes PDAC and CRC progression by inhibiting NF-κB activation.

In order to visualize the binding of PR5-LL-CM01 with PRMT5, we employed structural modeling approach using the existing information of PRMT5 crystal structure [26]. PR5-LL-CM01 was docked in the presence (SAM-bound) or absence of SAM (Apo-PRMT5). Under SAM-bound condition (Figure 7A, left panel), the binding sites for PR5-LL-CM01 and EPZ015666 on PRMT5 overlap to a great extent, indicating that these two inhibitors probably interact with PRMT5 through largely similar group of binding sites. However, in the Apo-PRMT5 condition, though the binding sites of EPZ015666 remain the same as those in SAM-bound condition, the binding position of PR5-LL-CM01 shifted dramatically from its original binding position in the SAM-bound condition to the new position (pink dotted line, Figure 7A, right panel), which is, intriguingly, same as the SAM binding position in the SAM-bound condition (Figure 7A, left panel). This interesting phenomenon suggest that PR5-LL-CM01 could possibly interfere with the residues involved in SAM binding to PRMT5 in SAM-bound PRMT5, but can also block the binding of SAM to Apo-PRMT5 by occupying the similar binding site for SAM. In contrast, EPZ015666 binding stayed the same in both SAM-bound or Apo-PRMT5 conditions, thus binding to PRMT5 in a completely independent manner from SAM, which is consistent with previously published data [25]. In addition, we showed that both binding events under SAM-bound or Apo-SAM conditions are energetically favorable, with the Apo-PRMT5 (-7.911 kcal/mol) being slightly more favorable than the SAM-bound condition (-6.949 kcal/mol) (Figure 7B). A ligand binding affinity map (Figure 7C) further depicts the PRMT5 residues that interact with PR5-LL-CM01 in the SAM-bound condition. Importantly, PR5-LL-CM01 forms a hydrogen bond with the Glu444 residue in the PRMT5 active pocket. Since this residue is essential for the methyltransferase activity of PRMT5, this interaction points out yet another layer of important mechanism regarding how PR5-LL-CM01 may inhibit PRMT5 activity in addition to what we have proposed in Figure 7A and 7B.

As described in Figure 7A, under the SAM-bound condition, PR5-LL-CM01 and EPZ015666 bind to PRMT5 in a largely overlapped region. To look at these sites in much more detail, we summarized these interactive residues in Figure 7D. The sites that can solely interact with PRMT5 by PR5-LL-CM01 are denoted in red, solely by EPZ015666 in blue, or by both in green. Most of the residues are present in the Rossman fold, followed by several residues in the β-barrel, and some residues in the linker domain of the PRMT5 structure. Since the methyltransferase domain is comprised within the Rossman and β-barrel domains, these evidences further support that PR5-LL-CM01 binds to residues in the catalytic domain of PRMT5. We show that in addition to a few commonly shared binding sites with EPZ015666, PR5-LL-CM01 largely exhibits its quite unique binding sites to PRMT5 (Figure 7D).

Taken together, we hypothesize that PRMT5 overexpression can promote the cancer phenotype through PRMT5-mediated NF-κB activation, whereas using PR5-LL-CM01 to block this activity could impede PDAC and CRC progression (Figure 7E). Therefore, PR-LL-CM01 could serve as the drug development basis to treat PDAC and CRC in the very near future.

## DISCUSSION

The White House Cancer Moonshot's mission is to double the rate of progress in cancer research and treatment. Both PDAC and CRC are among the leading cause of cancer-related deaths in the United States. Despite important advances in recent years, many patients continue to experience disease recurrence following primary therapy. Moreover, there is currently “no cure” for a significant number of patients presenting with metastasis upon diagnosis. An estimated $20 billion will be spent on PDAC and CRC care in the United States in 2016, reflecting the urgent medical need for the discovery of newer PDAC and CRC treatment options.

Post-translational modifications (PTMs) regulate protein function in eukaryotes and have been shown to play important roles in a variety of cancers [38]. Methylation of lysine and arginine is one of the most critical PTMs seen in nature. In this study, we are the first to show that PRMT5 expression was significantly upregulated in PDAC and CRC. Furthermore, increased expression of PRMT5 in cancer cells led to enhanced cancer-promoting characteristics, including cell proliferation, anchorage-independent growth, and cell migration, clearly highlighting the role of PRMT5 as a tumor promoter in PDAC and CRC.

NF-κB is a critical transcription factor that is hyperactivated in various cancers. Since it also plays a crucial role in normal cellular functioning, direct targeting of NF-κB has not proven to be a successful therapeutic approach [39]. These obstacles highlight the importance of identifying pathway-specific inhibition of NF-κB activity in cancer treatment. In this regard, the discovery of PRMT5-mediated activation of NF-κB and its targeted inhibition in PDAC and CRC is of great importance and significance. Furthermore, the successful development and application of the PRMT5-specific AlphaLISA HTS technique in this study constitutes another layer of unique contribution to the drug discovery field. Using this sensitive approach, we successfully identified PR5-LL-CM01 as our leading hit and confirmed that it is a highly potent and specific PRMT5 inhibitor. Using a closely related structural analog, PR5-LL-IEC01, we demonstrated the specificity of the effect shown by PR5-LL-CM01. Currently, EPZ015666 is the only specific PRMT5 inhibitor available on the market [25]. Chan *et al*. reported that EPZ015666 is very effective in inhibiting mantle cell lymphoma [25]. We showed that PR5-LL-CM01 was ~10-15 fold more potent than EPZ015666 in PDAC and CRC models, making PR5-LL-CM01 the first PRMT5 inhibitor to be highly effective in the treatment of solid tumors. We speculate that PR5-LL-CM01 could be more effective than EPZ015666 in the treatment of other solid cancers with hyper PRMT5 expression. We are very eager to test this possibility in the very near future.

Additionally, structural modeling experiments showed that PR5-LL-CM01 and EPZ015666 could interact with PRMT5 through quite different mechanisms. Particularly, PR5-LL-CM01 interacts with Glu444 on PRMT5, a critical residue for the catalytic activity of PRMT5 [26]. Interestingly, we observed that all the residues participating in the binding interactions between PR5-LL-CM01 and PRMT5 span over the Rossman fold and β-barrel domains of PRMT5, which have been previously shown to comprise the catalytic methyltransferase domain of PRMT5 and the region where SAM and its substrates bind [26]. On the other hand, EPZ015666 binds to PRMT5 independent from SAM binding sites. These quite different PRMT5 binding mechanisms could shed light on why PR5-LL-CM01 is more potent than EPZ015666 in killing PDAC and CRC cells.

Of note, it is possible that PRMT5-mediated pathways are complicated. Though many mechanisms of the tumor-promoting tendencies of PRMT5 are still unclear, the insights provided by this study are extremely valuable in understanding the workings of PRMT5. They could also possibly be applied to other cancers beyond PDAC and CRC. PRMT5 is upregulated in various types of cancers, including but not limited to pancreas, colon, breast, prostate, and lung cancers, as well as lymphoma and melanoma [40–44]. Due to the commonality of high levels of PRMT5, our current findings with regards to PDAC and CRC could have broader impacts on the understanding of cancer progression as a whole.

In summary, we have identified the significant role of PRMT5 as a tumor promoter in PDAC and CRC, as well as highlighted its potential to be exploited as an important therapeutic target. Building upon this exciting work, our immediate future plan would be to generate derivatives of PR5-LL-CM01 with the ultimate goal of moving this discovery to clinical application. In terms of how PR5-LL-CM01 inhibits PRMT5, one line of research we would like to pursue is to further verify our *in silico* model with purified PRMT5 crystal for the structure analysis. We will also determine the direct binding of PR5-LL-CM01 and its derivatives to PRMT5 with isothermal calorimetry (ITC) experiments, a cutting-edge technique that is able to monitor direct binding of a ligand to a protein.

Overall, the discovery of PR5-LL-CM01 opens a door to promising new PDAC and CRC therapies and gateways to better understanding the workings behind PDAC and CRC as a whole. In the long run, we will move toward clinical trials with the few best-characterized PR5-LL-CM01 derivatives with the ultimate goal of improving PDAC and CRC patient survivorship and treatment.

## MATERIALS AND METHODS

### Cell lines

Pancreatic control (HPNE) and cancer cell lines (PANC1, MiaPaCa2 and AsPC1) were a kind gift from Dr. Murray Korc (Indiana University School of Medicine). The normal colon cell line (FHC) and CRC (HT29, HCT116, DLD1) cell lines were purchased from the American Tissue Culture Collection. All pancreas-derived cell lines were grown in Dulbecco's Modified Eagle Medium (DMEM) (GE Healthcare), supplemented with 1% of penicillin/streptomycin, 10% fetal bovine serum (FBS). CRC cells were maintained in Roswell Park Memorial Institute Medium (RPMI 1640) (GE Healthcare), containing 1% penicillin/streptomycin and 10% FBS, while FHC cells were cultured under the same condition with further addition of 25mM HEPES (4-(2-hydroxyethyl)-1-piperazineethanesulfonic acid), 10ng/ml cholera toxin, 0.005 mg/ml insulin, 0.005 mg/ml transferrin and 100 ng/ml hydrocortisone. All cell lines were cultured at 37°C under 5% CO_2_ and used between passages 2 to 3. Cell lines were authenticated using 9 Marker STR Profile.

### Western blotting

Protein samples were run on a 10% polyacrylamide gel and then transferred overnight to a polyvinylidene fluoride (PVDF) membrane (Fisher Scientific) at 4°C. Primary antibodies for anti-PRMT5 (Abcam; ab109451), anti-Flag (Sigma-Aldrich; F1804), anti-dimethyl arginine motif (sdme-RG) (Cell Signaling Technology; 13222) and β-actin (Sigma-Aldrich; A5316) and their corresponding secondary antibodies were used. The enhanced chemiluminescent (ECL) detection method (PerkinElmer) was conducted to detect the protein signal.

### Immunohistochemistry assay

Pancreatic and colon cancer tissue microarrays with matched normal adjacent controls were acquired from US Biomax Inc. The tissue microarrays were blocked using protein-blocking solution (Dako Corp.) for 30 min. All subsequent staining steps were performed using the Dako FLEX SYSTEM and an automated Immunostainer. Incubations were carried out at room temperature and Tris buffered saline containing 0.05% Tween 20, pH 7.4 (Dako Corp.) was used for all the washes and diluents. Anti-PRMT5 primary antibody (Abcam; ab109451) was used to detect PRMT5 localization. Horseradish peroxidase-conjugated secondary antibody was then used, followed by addition of the chromogen, which formed a brown precipitate at the binding site of secondary antibody. Imaging was done using Aperio whole slide digital imaging system. The system imaged all slides at 20X magnification.

### Luciferase assay

The NF-κB luciferase construct p5XIP10 (containing five tandem copies of the NF-κB site from the IP10 gene) [21] was transfected transiently in the cell lines using Lipofectamine and PLUS Reagents (Invitrogen). Luciferase activity was assayed 48 hrs later by using the Luciferase Assay System with Reporter Lysis Buffer kit (Promega) per the manufacturer's instructions. The activity was measured using a Synergy H1 Multi-Mode Reader (BioTek Instruments Inc.).

### Anchorage-independent assay

2.4% and 1.25% agar were used to prepare the bottom and top layers of the soft agar, respectively. The bottom agar was added to each well of a 6-well plate. 2×10^5^ cells for each cell line were then mixed into top agar solution and layered on top of the bottom layer. The plates were incubated for 10-20 days at 37°C and 5% CO_2_. Images were captured using a Canon EOS Rebel T3i Digital SLR camera and quantification of colony size and number was performed using ImageJ.

### Migration assay

Migration assay was conducted using Boyden chambers. 8μm pore size cell culture inserts (Corning) were placed in a 24-well plate. Each chamber was coated with gelatin on the side facing the lower chamber. 2×10^5^ cells were suspended in serum-starved media in the upper chamber of the well. Corresponding serum rich media was added to the lower chamber. Migrated cells were fixed using 4% formaldehyde followed by crystal violet staining and counting using a microscope at 20X magnification. The images were captured using a Canon EOS Rebel T3i Digital SLR camera.

### AlphaLISA HTS screening

PRMT5 enzyme was purified with anti-Flag-M2 beads (Sigma-Aldrich) from 293 cells with the overexpression of Flag-PRMT5 protein, as described previously [21]. The enzyme prep was diluted in assay buffer (30mM Tris, pH 8.0, 1mM DTT, 0.01% BSA, 0.01% Tween-20) before use. SAM (New England Biolabs) was used as the methyl group donor and unmethylated histone H4R3 (Anaspec) was used as a substrate. For screening, library compounds were added with the robotic system. All these components were incubated at room temperature (R.T.) for 1 hr. Acceptor beads and Donor beads were then diluted in 1X Epigenetics buffer (PerkinElmer) before use. Acceptor beads were then added at a final concentration of 20 μg/ml to the reaction mixture and the plate was incubated at R.T. for 1 hr. Donor beads were added at a final concentration of 20 μg/ml and the plate was incubated at R.T. for 30 min. The reaction was run in 384-well plates. The plates were read using an EnVision^®^ Reader.

### Co-immunoprecipitation experiment

293 with stable overexpression of Flag-p65 protein, were treated with 20μM of PR5-LL-CM01 for 24 hrs. p65 was then pulled down with anti-Flag-M2 beads, using immunoprecipitation methods as described previously [21]. The samples were then run on a 10% protein gel, and probed with anti-dimethyl arginine motif (sdme-RG) antibody (Cell Signaling Technology; 13222), to check for dimethylation levels of p65.

### MTT [(3-(4, 5-dimethylthiazolyl-2)-2, 5-diphenyltetrazolium bromide)] assay

Cells were seeded at 90% confluence in 96-well plates and titrated with different dosages of PR5-LL-CM01 for 4 days. Media was then removed and 10μl of MTT (Sigma-Aldrich) was added per well. Cells were incubated for 2 hrs at 37°C before adding 100μl of DMSO to each well and quantified with the Synergy H1 Multi-Mode Reader (BioTek Instruments Inc).

### Quantitative PCR

Cells were cultured to 90% confluence and total RNA was isolated using Trizol as described previously [21]. cDNA was prepared by reverse-transcriptase PCR from total RNA using the SuperScript III First-Strand Synthesis System (Invitrogen). qPCR was carried out using FastStart Universal SYBR Green Master ROX (Roche). Primers were designed by Primer Express 3.0 software and will be available upon request.

### Tumor efficacy study

Male NSG mice were obtained from the *In Vivo* Therapeutics Core at Indiana University School of Medicine. After acclimation for 7 days, NSG mice (6-8 weeks old) were xenografted with Mycoplasma-free PANC1 or HT29 cells subcutaneously (1 × 10^7^ PANC1 or 3 × 10^6^ HT29 cells used per mouse in 0.2 ml of a 1:1 mix of phosphate-buffered saline and Matrigel) (BD Biosciences). 5 mice were randomized in each group when tumor volumes reach ~100mm^3^. Mice were treated with either vehicle control or 20mg/kg of PR5-LL-CM01 (drug stock dissolved in 1:1 Cremophor:ethanol solution) intraperitoneally 3 times per week. Tumor volumes and body weights were measured twice a week. The study was performed in accordance with the guidelines and standards of the Institutional Animal Care and Use Committee (IACUC) and under the approved animal protocol # 11066 MD/R by Indiana University.

### Structural analysis and docking experiments

For docking, chain A of PRMT5 protein in 4×61.pdb from the Protein Databank was used. All docking experiments were performed by Glide program (version 6.8) of the Schrodinger suite 2015-3 in standard precision mode [27, 28]. The protein was prepared for docking by the Protein Preparation Wizard of Schrodinger suite 2015-3 [29]. Control docking experiment was performed by deleting the ligand PR5-LL-CM01 or EPZ015666 from the crystal structure while keeping the ligand SAM. The control docking experiment was able to reproduce the binding pose of EPZ015666 seen in the crystal structure. An additional docking experiment was done by docking EPZ015666 into the PRMT5 active site after deleting the SAM ligand as well as EPZ015666 from the active site. PR5-LL-CM01 was docked into the PRMT5 active site in two separate docking experiments. In one experiment, the compound was docked into the PRMT5 active site in which EPZ015666 is deleted while SAM is kept. In the other experiment, PR5-LL-CM01 was docked into the completely empty active site in which SAM was deleted. All the figures for this experiment were prepared with Maestro version 10.3 [30].

### Statistical analysis

Statistical analysis was performed using Prism 6 software (GraphPad, San Diego, CA). Results have been presented as mean ± SD or mean ± SEM, as specified. A two-tailed Student's t test was used while comparing two means to test for significant differences. All statistics were calculated on triplicate experiments and *p* value < 0.05 was considered statistically significant.

## SUPPLEMENTARY MATERIALS FIGURES AND TABLE



## References

[1] Siegel R, Naishadham D, Jemal A (2013). Cancer statistics, 2013. CA Cancer J Clin.

[2] Hidalgo M (2010). Pancreatic cancer. N Engl J Med.

[3] Conroy T, Desseigne F, Ychou M, Bouche O, Guimbaud R, Becouarn Y, Adenis A, Raoul JL, Gourgou-Bourgade S, de la Fouchardiere C, Bennouna J, Bachet JB, Khemissa-Akouz F (2011). FOLFIRINOX versus gemcitabine for metastatic pancreatic cancer. N Engl J Med.

[4] Ferlay J, Soerjomataram I, Dikshit R, Eser S, Mathers C, Rebelo M, Parkin DM, Forman D, Bray F (2015). Cancer incidence and mortality worldwide: sources, methods and major patterns in GLOBOCAN 2012. Int J Cancer.

[5] Daniluk J, Liu Y, Deng D, Chu J, Huang H, Gaiser S, Cruz-Monserrate Z, Wang H, Ji B, Logsdon CD (2012). An NF-κB pathway-mediated positive feedback loop amplifies Ras activity to pathological levels in mice. J Clin Invest.

[6] Ling J, Kang Y, Zhao R, Xia Q, Lee DF, Chang Z, Li J, Peng B, Fleming JB, Wang H, Liu J, Lemischka IR, Hung MC (2012). KrasG12D-induced IKK2/beta/NF-κB activation by IL-1alpha and p62 feedforward loops is required for development of pancreatic ductal adenocarcinoma. Cancer Cell.

[7] Fujioka S, Sclabas GM, Schmidt C, Frederick WA, Dong QG, Abbruzzese JL, Evans DB, Baker C, Chiao PJ (2003). Function of NF-κB in pancreatic cancer metastasis. Clin Cancer Res.

[8] Wang W, Abbruzzese JL, Evans DB, Larry L, Cleary KR, Chiao PJ (1999). The NF-κB RelA transcription factor is constitutively activated in human pancreatic adenocarcinoma cells. Clin Cancer Res.

[9] Sakamoto K, Maeda S, Hikiba Y, Nakagawa H, Hayakawa Y, Shibata W, Yanai A, Ogura K, Omata M (2009). Constitutive NF-κB activation in colorectal carcinoma plays a key role in angiogenesis, promoting tumor growth. Clin Cancer Res.

[10] Voboril R, Weberova-Voborilova J (2006). Constitutive NF-κB activity in colorectal cancer cells: impact on radiation-induced NF-κB activity, radiosensitivity, and apoptosis. Neoplasma.

[11] Mundade R, Imperiale TF, Prabhu L, Loehrer PJ, Lu T (2014). Genetic pathways, diagnosis and treatment in sporadic colon cancer. Oncoscience.

[12] Lu T, Sathe SS, Swiatkowski SM, Hampole CV, Stark GR (2004). Secretion of cytokines and growth factors as a general cause of constitutive NF-κB activation in cancer. Oncogene.

[13] Ghosh S, May MJ, Kopp EB (1998). NF-κB and Rel proteins: evolutionarily conserved mediators of immune responses. Annu Rev Immunol.

[14] Prabhu L, Mundade R, Korc M, Loehrer PJ, Lu T (2014). Critical role of NF-κB in pancreatic cancer. Oncotarget.

[15] Liou GY, Doppler H, Necela B, Krishna M, Crawford HC, Raimondo M, Storz P (2013). Macrophage-secreted cytokines drive pancreatic acinar-to-ductal metaplasia through NF-κB and MMPs. J Cell Biol.

[16] Agarwal A, Das K, Lerner N, Sathe S, Cicek M, Casey G, Sizemore N (2005). The AKT/I-κB kinase pathway promotes angiogenic/metastatic gene expression in colorectal cancer by activating nuclear factor-kappa B and beta-catenin. Oncogene.

[17] Yu HG, Zhong X, Yang YN, Luo HS, Yu JP, Meier JJ, Schrader H, Bastian A, Schmidt WE, Schmitz F (2004). Increased expression of NF-κB/RelA is correlated with tumor angiogenesis in human colorectal cancer. Int J Colorectal Dis.

[18] Gilmore TD (2006). Introduction to NF-NF-κB: players, pathways, perspectives. Oncogene.

[19] Arora S, Bhardwaj A, Singh S, Srivastava SK, McClellan S, Nirodi CS, Piazza GA, Grizzle WE, Owen LB, Singh AP (2013). An undesired effect of chemotherapy: gemcitabine promotes pancreatic cancer cell invasiveness through reactive oxygen species-dependent, NF-κB- and hypoxia-inducible factor 1alpha-mediated up-regulation of CXCR4. J Biol Chem.

[20] Lind DS, Hochwald SN, Malaty J, Rekkas S, Hebig P, Mishra G, Moldawer LL, Copeland EM, Mackay S (2001). NF-κB is upregulated in colorectal cancer. Surgery.

[21] Wei H, Wang B, Miyagi M, She Y, Gopalan B, Huang DB, Ghosh G, Stark GR, Lu T (2013). PRMT5 dimethylates R30 of the p65 subunit to activate NF-κB. Proc Natl Acad Sci U S A.

[22] Mundade R, Wei H, Lu T (2014). PRMT5, a pivotal player in cancer. Austin J Pharm & Toxicol.

[23] Wei H, Mundade R, Lange KC, Lu T (2014). Protein arginine methylation of non-histone proteins and its role in diseases. Cell Cycle.

[24] Lu T, Stark GR (2015). NF-κB Regulation by Methylation. Cancer Res.

[25] Chan-Penebre E, Kuplast KG, Majer CR, Boriack-Sjodin PA, Wigle TJ, Johnston LD, Rioux N, Munchhof MJ, Jin L, Jacques SL, West KA, Lingaraj T, Stickland K (2015). A selective inhibitor of PRMT5 with in vivo and in vitro potency in MCL models. Nat Chem Biol.

[26] Antonysamy S, Bonday Z, Campbell RM, Doyle B, Druzina Z, Gheyi T, Han B, Jungheim LN, Qian Y, Rauch C, Russell M, Sauder JM, Wasserman SR (2012). Crystal structure of the human PRMT5: MEP50 complex. Proc Natl Acad Sci U S A.

[27] Halgren TA, Murphy RB, Friesner RA, Beard HS, Frye LL, Pollard WT, Banks JL (2004). Glide: a new approach for rapid, accurate docking and scoring. 2. Enrichment factors in database screening. J Med Chem.

[28] Friesner RA, Banks JL, Murphy RB, Halgren TA, Klicic JJ, Mainz DT, Repasky MP, Knoll EH, Shelley M, Perry JK, Shaw DE, Francis P, Shenkin PS (2004). Glide: a new approach for rapid, accurate docking and scoring. 1. Method and assessment of docking accuracy. J Med Chem.

[29] Sastry GM, Adzhigirey M, Day T, Annabhimoju R, Sherman W (2013). Protein and ligand preparation: parameters, protocols, and influence on virtual screening enrichments. J Comput Aided Mol Des.

[30] (2015). Schrödinger Release 2015-3: Maestro, version 10.3.

[31] Shaw LM (2005). Tumor cell invasion assays. Methods Mol Biol.

[32] Simard JR, Plant M, Emkey R, Yu V (2013). Development and implementation of a high-throughput AlphaLISA assay for identifying inhibitors of EZH2 methyltransferase. Assay Drug Dev Technol.

[33] Quinn AM, Allali-Hassani A, Vedadi M, Simeonov A (2010). A chemiluminescence-based method for identification of histone lysine methyltransferase inhibitors. Mol Biosyst.

[34] Zhang JH, Chung TD, Oldenburg KR (1999). A Simple Statistical Parameter for Use in Evaluation and Validation of High Throughput Screening Assays. J Biomol Screen.

[35] Horiuchi KY, Eason MM, Ferry JJ, Planck JL, Walsh CP, Smith RF, Howitz KT, Ma H (2013). Assay development for histone methyltransferases. Assay Drug Dev Technol.

[36] Fennema E, Rivron N, Rouwkema J, van Blitterswijk C, de Boer J (2013). Spheroid culture as a tool for creating 3D complex tissues. Trends Biotechnol.

[37] Heylman C, Sobrino A, Shirure VS, Hughes CC, George SC (2014). A strategy for integrating essential three-dimensional microphysiological systems of human organs for realistic anticancer drug screening. Exp Biol Med (Maywood).

[38] Karve TM, Cheema AK (2011). Small changes huge impact: the role of protein posttranslational modifications in cellular homeostasis and disease. J Amino Acids.

[39] Martin M, Wei H, Lu T (2016). Targeting microenvironment in cancer therapeutics. Oncotarget.

[40] Jiang W, Newsham IF (2006). The tumor suppressor DAL-1/4.1B and protein methylation cooperate in inducing apoptosis in MCF-7 breast cancer cells. Mol Cancer.

[41] Gu Z, Gao S, Zhang F, Wang Z, Ma W, Davis RE, Wang Z (2012). Protein arginine methyltransferase 5 is essential for growth of lung cancer cells. Biochem J.

[42] Cho EC, Zheng S, Munro S, Liu G, Carr SM, Moehlenbrink J, Lu YC, Stimson L, Khan O, Konietzny R, McGouran J, Coutts AS, Kessler B (2012). Arginine methylation controls growth regulation by E2F-1. EMBO J.

[43] Zhang HT, Zhang D, Zha ZG, Hu CD (2014). Transcriptional activation of PRMT5 by NF-Y is required for cell growth and negatively regulated by the PKC/c-Fos signaling in prostate cancer cells. Biochim Biophys Acta.

[44] Wang L, Pal S, Sif S (2008). Protein arginine methyltransferase 5 suppresses the transcription of the RB family of tumor suppressors in leukemia and lymphoma cells. Mol Cell Biol.

