# Supplementary material for: Adapting AlphaLISA high throughput screen to discover a novel small-molecule inhibitor targeting protein arginine methyltransferase 5 in pancreatic and colorectal cancers
Source: Oncotarget. 2017 May 23;8(25):39963–77. doi: 10.18632/oncotarget.18102 (PMC5522311; doi:10.18632/oncotarget.18102)
Supplement: Supplementary file 1 [file oncotarget-08-39963-s001.pdf]

Adapting AlphaLISA high throughput screen to discover a novel small-molecule inhibitor targeting protein arginine methyltransferase 5 in pancreatic and colorectal cancers

Supplementary Materials

METHODS

Methyltransferase assay

The specificity of inhibition by PR5-LL-CM01 against the enzymatic activity of protein arginine methyltransferase family members was analyzed using the HotSpot radioisotope-based platform (Reaction Biology Corp) as described previously [35]. Briefly, PR5-LL-CM01 was incubated with a protein arginine methyltransferase, substrate, and tritium-labeled SAM. The PR5-LL-CM01 stock solution was prepared in DMSO at 10mM. PR5-LL-CM01 was tested at 1, 10, 25, 50 and 100µM. The methyltransferase inhibitor SAH (S-(5'-Adenosyl)-L-homocysteine) was used as a positive control. All reactions were carried out with 1µM tritium-labeled SAM and 5µM peptide substrate. The assays were performed using Reaction buffer (50mM Tris-HCl, pH 8.5, 5mM $MgCl_2$, 50mM NaCl, 0.01% Brij35, 1mM DTT). Reactions were performed for 1h at 30°C. Radiolabeled methylated product was detected using a filter-binding method. Curve fits and IC_{50} determination were executed as described previously [35].

3D colony formation assay

500 cells per well were seeded in media containing 3% Reduced Growth Factor (RGF)-Matrigel (BD Biosciences) in a Corning® Costar® ultra-low attachment multiwell plate and allowed to form colonies. PR5-LL-CM01 stocks made in 100% DMSO were diluted using 3% RGF-Matrigel media and added to the respective wells, in a 2-fold serial dilution concentration range from 0.75 to 25 µM on days 3 and 7 after seeding. On day 7 post first drug treatment, the cells were stained using Alamar blue dye (Fisher), and quantified using a Synergy H4 Multi-Mode Reader (BioTek Instruments Inc).

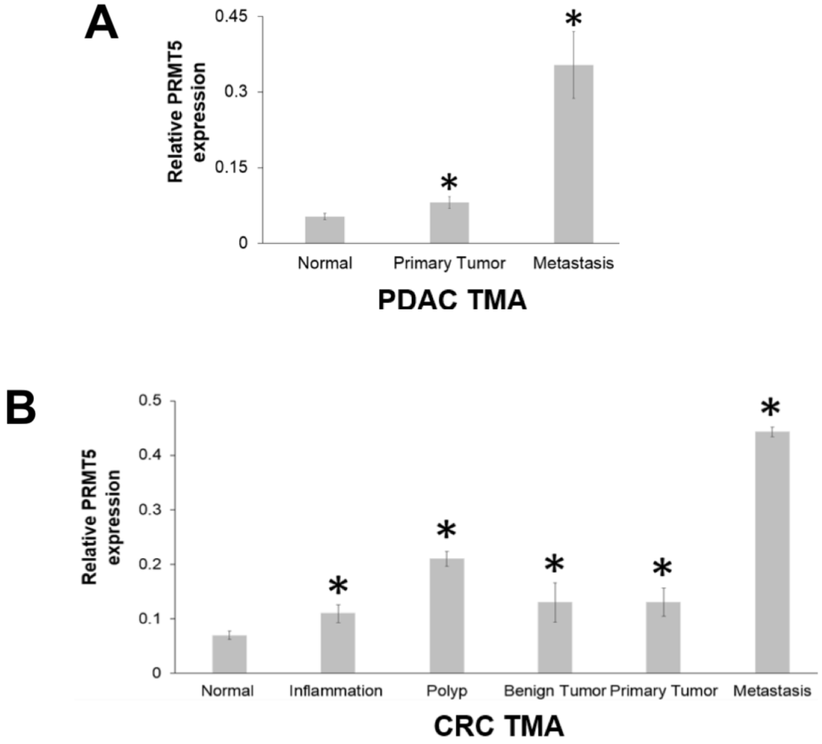

Supplementary Figure 1. Quantification for immunohistochemical analysis of the PDAC and CRC tumor microarray (TMA). A. Bar graph showing a significant increase in relative PRMT5 expression in primary stage tumor (n=19) and metastatic tumor patient tissue (n=6) vs. normal tissue (n=24) for the PDAC TMA. *p< 0.05, different samples *vs.* normal control. B. IHC quantification of CRC TMA show a similar increase in relative PRMT5 expression for the representative disease stages in the TMA, including inflammation (n=9), polyp (n=5), benign tumors (n=5), primary tumors (n=14) and metastatic tissue (n=20) vs. normal patient tissue (n=9). *p< 0.05, different samples *vs.* normal control.

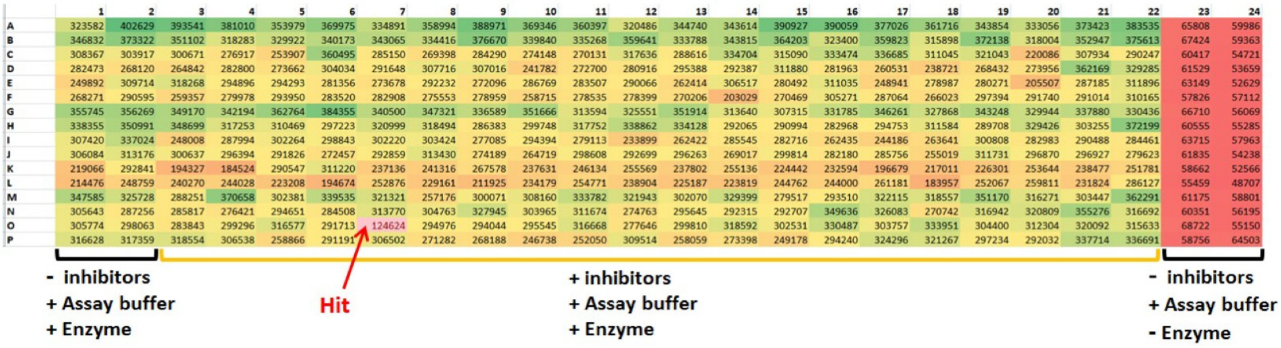

Supplementary Figure 2. Representation of a 384-well plate used in the HTS, with positive control (no inhibitor, with enzyme), test compounds (inhibitor and enzyme present) and negative controls (no inhibitor, no enzyme). A potential hit with decreased AlphaLISA signal is indicated with a red arrow.

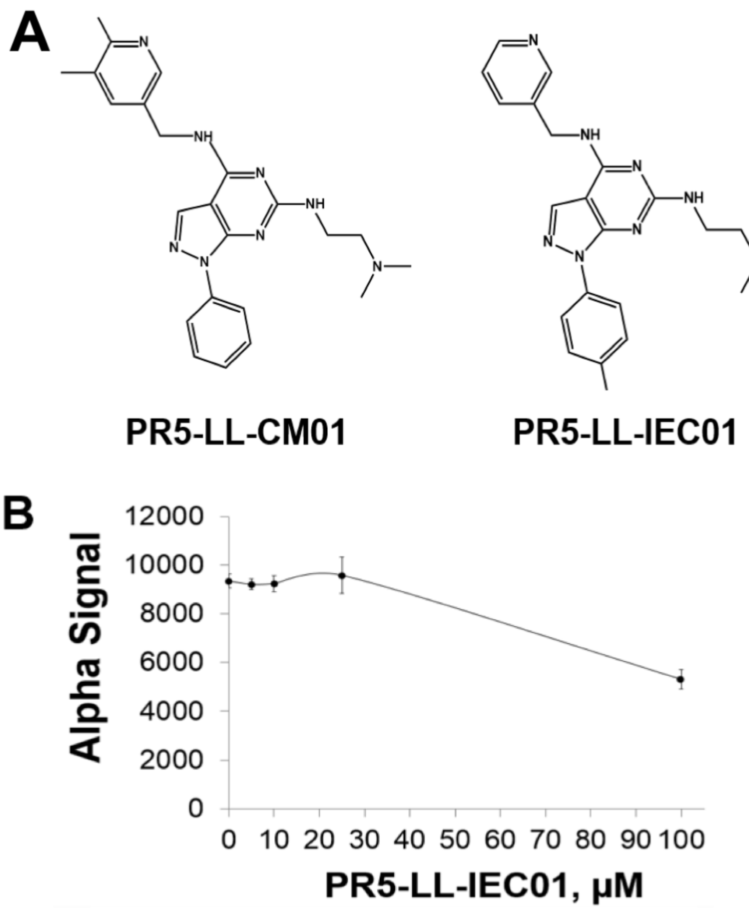

Supplementary Figure 3. PR5-LL-IEC01, a structural analog of PR5-LL-CM01. A. Structure of PR5-LL-IEC01, in side-by-side comparison to the lead compound, PR5-LL-CM01. B. Calculation of IC_{50} of PR5-LL-IEC01 using AlphaLISA, with IC_{50} calculated to be ~118μM, ~16-fold higher than the lead compound PR5-LL-CM01.

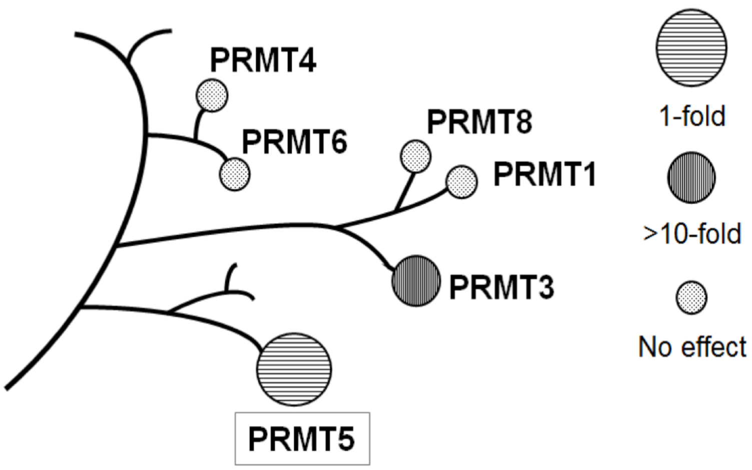

Supplementary Figure 4. Methyltransferase assay. The specificity of inhibition by PR5-LL-CM01 against the enzymatic activity of protein arginine methyltransferase family members was analyzed using the HotSpot radioisotope-based platform (Reaction Biology Corp) [35]. PR5-LL-CM01 showed high specificity to PRMT5, while showed either zero effect or at least a 10-fold higher IC_{50} to other PRMT family members than that of PRMT5.

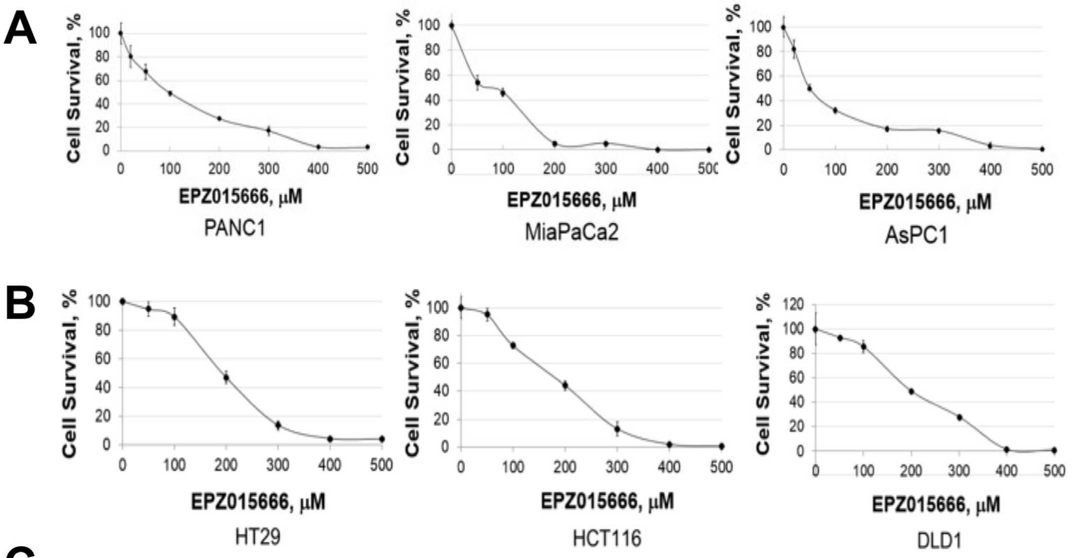

C

Cancer Type	Cell line	IC50, µM
PDAC	PANC1	95
	MiaPaCa2	62
	AsPC1	50
CRC	HT29	190
	HCT116	180
	DLD1	195

Supplementary Figure 5. Effect of EPZ015666 on PDAC and CRC lines. A. MTT assay, showing that in PDAC cells (PANC1, MiaPaCa2 and AsPC1) EPZ015666 decreased cell viability, however had a lower efficacy to decrease cell viability than that of PR5-LL-CM01 (Figure. 4A). B. MTT assay, showing that in CRC cells (HT29, HCT116, and DLD1), EPZ015666 decreased cell viability, but had lower efficacy to decrease cell viability than that of PR5-LL-CM01 (Figure 4B). The data represent the means ± S.D. for three independent experiments. *P < 0.05 *vs.* Ctrl group. C. Table summarizing the IC_{50} values for EPZ015666 in PDAC and CRC cell lines, respectively.

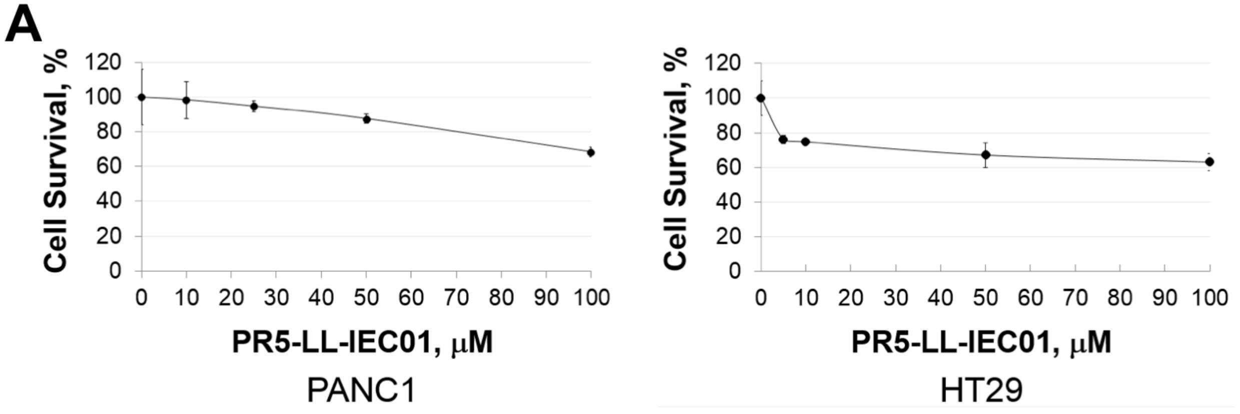

B

Cell Lines	PR5-LL-CM01 IC_{50}, µM	PR5-LL-IEC01 IC_{50}, µM
PANC1	4	161
HT29	10	138

Supplementary Figure 6. Effect of PR5-LL-IEC01 on cell viability of PDAC and CRC lines. A. MTT assay in PDAC (PANC1) and CRC (HT29), showing a high IC_{50} for PR5-LL-IEC01 in both the cell lines. B. Table, summarizing the IC_{50} values for PR5-LL-IEC01 in PANC1 and HT29 cells, respectively.

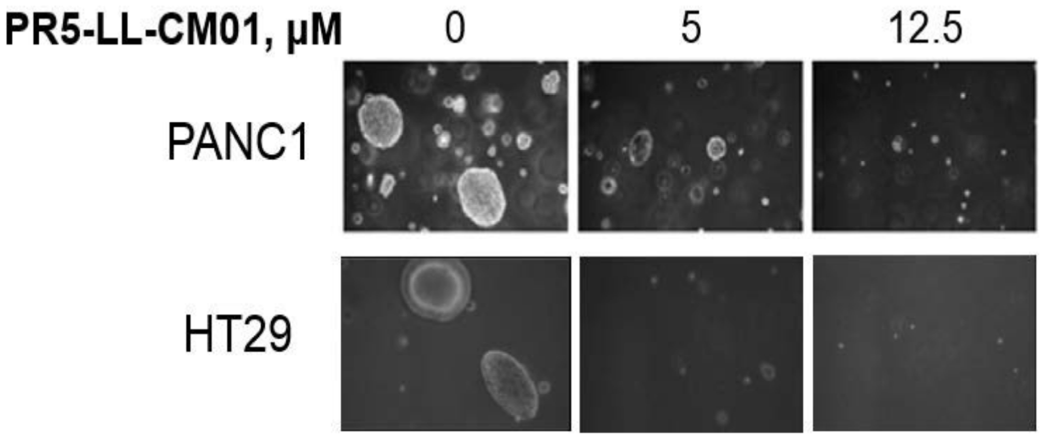

Supplementary Figure 7. Effect of PR5-LL-CM01 on anchorage-independent growth of PDAC and CRC lines. Anchorage-independent assay, showing that with increasing concentrations of PR5-LL-CM01, there was a significant decrease in the anchorage-independent growth ability in both PANC1 as well as HT29 cells.

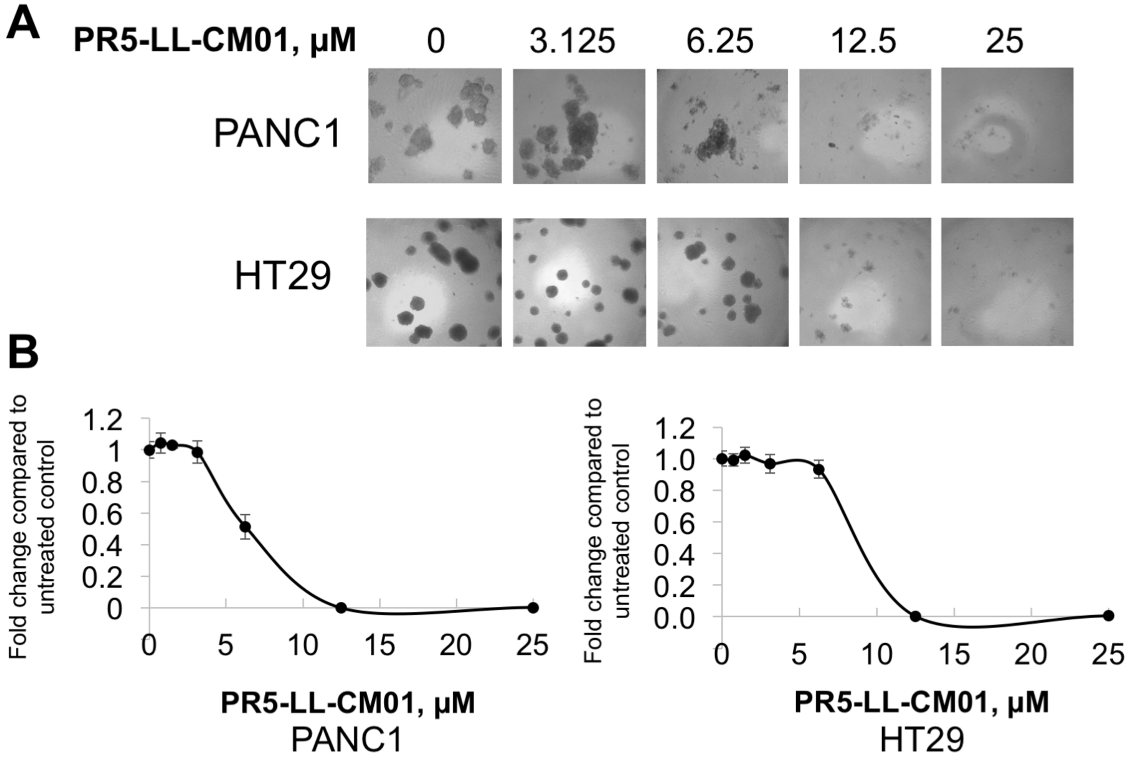

Supplementary Figure 8. Effect of PR5-LL-CM01 on 3D colony formation of PDAC and CRC lines. 3D colony formation assay, showing that with increasing concentrations of PR5-LL-CM01, there was a significant decrease in the 3D colony formation ability in both PANC1 as well as HT29 cells. A. Representative pictures in 4X magnification. B. Quantification for the fold change in 3D colony formation, upon treatment with increasing concentrations of PR5-LL-CM01, as compared to the untreated control.

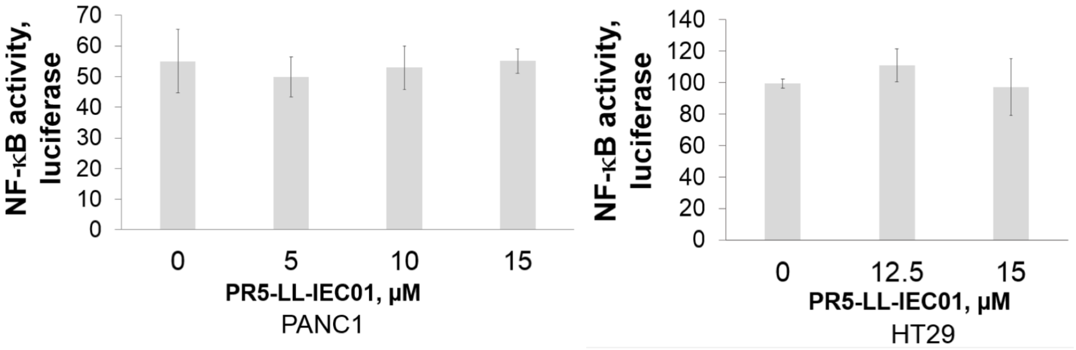

Supplementary Figure 9. Treatment with PR5-LL-IEC01 had no significant effect on NF-κB activation in PDAC and CRC cells. NF-κB luciferase assay, showing that upon treatment with increasing concentrations of PR5-LL-IEC01, there was no significant change in NF-κB activation in PANC1 and HT29 cells.

Supplementary Table 1: Table comparing the cancer cell lines with their respective normal control cells. The normal cells had a much higher cell survival rate at the IC_{50} observed in their cancer cell line counterparts.

Cancer	Cancer Cells	IC_{50}, μM	% survival in control cells (treated with IC_{50} of respective cancer cell line) PDAC normal control: HPNE CRC normal control: FHC
PDAC	PANC1	4	80%
	MiaPaCa2	2	90%
	AsPC1	4	80%
CRC	HT29	10	80%
	HCT116	10	80%
	DLD1	11	80%
